# Cannabinoids, Neurogenesis and Antidepressant Drugs: Is there a Link?

**DOI:** 10.2174/1570159X11311030003

**Published:** 2013-05

**Authors:** Manoela Viar Fogaça, Ismael Galve-Roperh, Francisco Silveira Guimarães, Alline Cristina Campos

**Affiliations:** 1 Department of Pharmacology; School of Medicine of RibeirãoPreto- University of São Paulo, Brazil; 2 Center for Interdisciplinary Research on Applied Neurosciences (NAPNA), University of São Paulo, Brazil; 3 Department of Biochemistry and Molecular Biology I, School of Biology, InstitutoUniversitario de Investigaciones Neuroquímicas (IUIN) and Instituto Ramón y Cajal de Investigación Sanitaria (IRYCIS), Complutense University, Spain; 4 Centro de Investigación Biomédica en Red sobre Enfermedades Neurodegenerativas (CIBERNED), Spain; 5 Group of Neuroimmunology, Laboratory of Immunopharmacology, Institute of Biological Sciences and School of Medicine, Federal University of Minas Gerais, Belo Horizonte, Brazil; 6 Infectious Diseases and Tropical Medicine Program, Medical School, Universidade Federal de Minas Gerais, Belo Horizonte, MG, Brazil

**Keywords:** Neurogenesis, antidepressant drugs, cannabinoids.

## Abstract

Similar to clinically used antidepressants, cannabinoids can also regulate anxiety and depressive symptoms. Although the mechanisms of these effects are not completely understood, recent evidence suggests that changes in endocannabinoid system could be involved in some actions of antidepressants. Chronic antidepressant treatment modifies the expression of CB_1_ receptors and endocannabinoid (EC) content in brain regions related to mood and anxiety control. Moreover, both antidepressant and cannabinoids activate mitogen-activated protein (MAP) kinase and phosphoinositide 3-kinase(PI3-K)/Akt or PKB signaling, intracellular pathways that regulate cell proliferation and neural cell survival. Facilitation of hippocampal neurogenesis is proposed as a common effect of chronic antidepressant treatment. Genetic or pharmacological manipulations of cannabinoid receptors (CB_1_ and CB_2_) or enzymes responsible for endocannabinoid-metabolism have also been shown to control proliferation and neurogenesis in the hippocampus. In the present paper we reviewed the studies that have investigated the potential contribution of cannabinoids and neurogenesisto antidepressant effects. Considering the widespread brain distribution of the EC system, a better understanding of this possible interaction could contribute to the development of therapeutic alternatives to mood and anxiety disorders.

## 1. ADULT NEUROGENESIS

Until the early 60´s, a central dogma of neuroscience had been that no new neurons are added to the adult mammalian brain. For more than 100 years it has been assumed that neurogenesis, or the production of new neurons, occurs only during development and stops before puberty. Although the very first reports about neurogenesis came from Dr Rita Levi-Montalcini’s work with Nerve Growth Factor, it was Joseph Altmanin the early 60´s that published a series of papers presenting evidence that new neurons are added in specific regions of the young and adult rat brain, including the neocortex, hippocampal formation and olfactory bulb [1-3]. Subsequently, Eriksson and colleagues (1998) confirmed that new neurons are indeed generated in the hippocampus of adult humans [4] and established one of the most stimulating recent fields in neuroscience: neurogenesis in the adult brain. 

Although a low proliferative activity has been reported in several brain regions such as the hypothalamus and the cell layers surrounding the third ventricle [5], a body of evidence supports the idea that in the adult mammalian brain only two regions show neurogenesis under physiological conditions: the subventricular zone (SVZ) of the lateral walls of the lateral ventricle and the subgranular zone (SGZ) of the dentate gyrus of the hippocampal formation [6, 7].

Adult neurogenesis is a complex process that involves the initial division of a precursor cells and lasts until the existence of a new functionally new neuron. In the words of Dr. G. Kempermann: *“neurogenesis is a process, not an event”. *It can be more precisely defined as an *in vivo* process that involves division, survival (not all dividing cells will survive), migration and differentiation [7, 8].

The physiological impact of adult neurogenesis is not yet completely understood. And importantly its relevance and existence in humans is matter of debate. SVZ neurogenesis seems to be regulated by the olfactory experience of animals [9, 10]. Odor exposure can increase the survival of newborn neurons and improve memory in a learned odor discrimination task [11], suggesting that in this region neurogenesis plays a role in learning and memory processes related to olfactory stimulation [11]. In the hippocampus SGZ, another major site of adult neurogenesis [12, 13], an association between this process and learning and memory has been found in rodents and humans [14-17]. Moreover, stimuli known to improve learning and memory processes, such as voluntary running and exposure to enriched environments [16, 18], increase SGZ cell proliferation and the survival of new neurons generated in this region [19, 20]. As a consequence, hippocampal neurogenesis has been suggested to be important for at least some forms of learning and memory [14-17]. Despite these pieces of evidence, adult neurogenesis is not necessarily always good to brain function. For example, increased neurogenesis after hippocampus injury could be involved in the development of temporal seizures [7].

The hippocampal formation is not an homogenous structure, showing differential connectivity along its dorsal-ventral (septum-temporal) axis. It has been proposed that, while the dorsal portions of hippocampus have a preferential role in learning and memory, the ventral portions of the hippocampus are involved in affective behaviors [21]. Also, several lines of evidence suggest that, in addition to learning and memory process, adult hippocampal neurogenesis could play an important role in the genesis of psychiatric disorders such as anxiety, schizophrenia and mood disorders [22-25]. In this way, stressful experiences, that are closely related to the development of anxiety and mood disorders, down-regulate hippocampal neurogenesis [26]. More recently, Snyder and colleagues (2011) showed that DG, but not SVZ neurogenesis, impairs stress-induced depressive-like symptoms and facilitates the negative hippocampal influence on the hypothalamic-pituitary-adrenal (HPA) axis [27]. Interestingly, drugs used in the clinical practice to treat these psychiatry disorders, such as antidepressants or lithium, normalize or even increase hippocampal neurogenesis [24, 28-30]. Together these findings support the proposal that adult hippocampal neurogenesis, in addition to influencing learning and memory process, is also involved in the genesis of psychiatry disorders and could, therefore, be a therapeutic target in these disorders.

## 2. NEUROGENESIS AND ANTIDEPRESSANTS

The mechanism of action of antidepressants (AD) has been the focus of a large number of studies in the last 50 years. Most of these studies were based on the monoaminergic theory of depression [31-37]. However, in the last decade, a neurogenic mechanism of action for AD opened new venues of investigation, particularly because the latency for antidepressants clinical effects (2-4weeks) coincides with the minimum time course necessary for the maturation of new neurons in the dentate gyrus [38]. Initial studies have showed that subchronic and chronic, but not acute, treatment with different classes of AD, such as fluoxetine (selective serotonin reuptake inhibitor, SSRI), imipramine (tricyclic, TC), reboxetine (norepinephrine reuptake inhibitor, NRI), tranylcypromine (monoamine oxidase inhibitor, MAOI), venlafaxine (serotonin-norepinephrine reuptake inhibitor, SNRI) and others increase adult hippocampal neurogenesis (see Table **1**) and, at the same time, cause antidepressive and anxiolytic effects and improvement of stress-disrupted responses [23, 28, 39]. 

In 2003 Santarelli and colleagues published a landmark study showing that some behavioral effects of AD depend on neurogenesis in the subgranular zone of the dentate gyrus [24]. Chronic treatment with fluoxetine and imipramine induced anxiolytic-like effects in the novelty suppressed feeding test in control mice but not in animals that were submitted to x-ray-irradiation of the SGZ (SGZ-x-irradiation), a procedure that blunts neurogenesis by killing cells undergoing proliferation. Since then, other studies using different animal models have corroborated these results [40-41]. However, it is unlikely that neurogenesis facilitation explains all the behavioral effects of AD. For example, chronic treatment with fluoxetine induces anxiolytic responses in BALBc/J mice without interfering in neurogenesis [42]. Moreover, mice submitted to the SGZ-x-irradiation or methylazoxymethanol, a cytostatic agent used to arrest neurogenesis, showed similar antidepressive responses to fluoxetine than control animals [43]. It is probable, therefore, that depending on the animal model and species used, multiple mechanisms are responsible for the effects of AD. 

Whereas most experimental data so far has suggested that a decrease of adult hippocampal neurogenesis is not directly responsible for depressive disorders [24, 40, 44] exposure to chronic stressors such as inescapable shocks, unpredictable stress, forced swim, social isolation and psychosocial conflict, decreases neuroproliferative processes in this brain region. Chronic AD treatment prevents this effect in different species such as rats, mice and primates [39, 40, 45-47]. In non-human primates, repeated social isolation, in addition to inducing depressive-like behaviors (anhedonia and subordinance), is also able to decrease cell proliferation and granule cell layer volume. Treatment with fluoxetine (15 weeks) prevented these effects in control animals but had no effect in SGZ-x-irradiated macaques, indicating neurogenesis-dependent action [47].

A question that remains open is how AD modulate neurogenesis. Most AD act by blocking monoamine uptake, and both serotonin and norepinephrine have been implicated in the increase of neuronal proliferation. A pioneer study showed that dl-fenfluramine, a compound that facilitates the release of 5-HT, promoted cellular division in the dentate gyrus, an effect that was blocked by the 5HT_1A_ receptor antagonist, WAY100,635 [48]. Also, administration of different 5HT_1A_ antagonists decreased the number of BrdU-immunoreactive cells in the dentate gyrus [49]. In 5HT_1A_ receptors knockout animals treated chronically with fluoxetine, both hippocampal neurogenesis and anxiolytic-like responses were abolished [24]. The deletion of 5HT_1A_ and 5HT_1B_ receptors decreased the expression of genes involved in long-term potentiation and adult neurogenesis and reduced hippocampal neurons survival [50]. Norepinephrine also stimulates cell division. It increases the proliferation of neural precursor derived cells, an effect that is blocked by selective β2-receptor antagonists [51]. Moreover, AD selectively increase nor epinephrine activated adult hippocampal precursors *via *β3-adrenergic receptors and β-adrenergic agonists enhanced nestin-GFAP positive neurons [52]. Finally, activation of 5-HT and β-adrenergic receptors influences the expression of important factors that modulate neuronal synaptic remodeling, proliferation, maturation and survival, including the brain derived neurotrophic factor (BDNF, [53]), the vascular endothelial growth factor (VEGF, [54]), proteins belonging to the cAMP-CREB cascade [54, 55], the Wnt3a signaling [56] and the p21 protein [57]. 

## 3. CANNABINOIDS AND NEUROGENESIS

### 3.1. Cannabinoids and the Endocannabinoid System

Cannabinoids were first extracted from the plant *Cannabis sativa*, which has at least 60 components that belong to this class of substances [58-63]. The observation that the activity of psychotropic cannabinoids was intrinsically related to its chemical structure [62, 63] led to the hypothesis that cannabinoid receptors exist in the organism. Subsequently, the cloning of CB_1_ and CB_2 _receptors confirmed their presence in rats, mice and humans (Howlett *et al*., 2002) and their activation inhibit the enzyme adenylatecyclase through a Gi/o protein [64-66].

CB_1 _receptors are now considered the most abundant metabotropic receptor in the mammalian brain and are also present in peripheral tissues [67]. Immunohistochemical evidence indicates that CB_1 _are located in several different adult brain regions, including those related to emotion and responses to aversive stimuli. They include the hippocampus [68, 69] striatum, *substantia nigra*, periaqueductal grey (PAG), amygdala, nucleus accumbens [69] and the cortex, especially the prefrontal cortex and cingulate [70, 71]. On the other hand, CB_2_ receptors are found mainly in cells of hematopoietic and immune system but are also present in the brain [72, 73]. 

Following the identification of these receptors various endogenous neuromodulators, named endocannabinoids (ECs), were discovered. Nowadays, the endocannabinoid (EC) system is proposed to comprise the CB_1_ and CB_2_ receptors, endogenous agonists derived from the arachidonic acid such as (N-arachidonoylethanolamide, AEA) and 2-arachidonoylglycerol (2-AG), and the proteins responsible for the synthesis and degradation of these molecules [74].

Although marijuana is considered a drug of abuse, some of its beneficial effects, including anticonvulsant, antipsychotic, antidepressant and anxiolytic actions, are due to its ability to regulate the endocannabinoid system [75-80]. Cannabinoids are able to alter brain activity by inhibiting calcium and activating potassium channels, resulting in inhibition of neurotransmitter release [81]. They can also promote neuronal plasticity, affecting short-term neuronal excitability by depolarization-induced suppression of inhibition (DSI), mainly in GABAergic synapses, and depolarization-induced suppression of excitation (DSE) in synapses governing the release of glutamate and the neuropeptide cholecystokinin [82-85]. Moreover, cannabinoids display neuroprotective actions, being involved in the control of glutamate-induced excitotoxicity [86-88]. In the last decade, other important mechanism of action of cannabinoids has been related to the improvement of emotional states: its regulatory role of adult hippocampal neurogenesis (see Table **2**).

### 3.2 Evidence from *in vitro* Studies

The EC system is present in the central nervous system since early stages of embryonic development and is involved in neuronal migration, survival and differentiation [89]. Embryonic neural progenitor cells (NPs) in culture express CB_1,_ CB_2 _receptors and FAAH. This is observed in cells that express nestin and incorporate BrdU, indicating that dividing cells express components of the EC system. Moreover, NPs can produce AEA and 2-AG, which are involved in the modulation of neuronal fate [90, 91]. Similar to the findings obtained in embryonic tissue, the EC system remains expressed and functional in adult stem/progenitor cells, inducing cell proliferation after cannabinoid challenge [92, 93]. 

NPs incubation with non-selective cannabinoid agonists such as AEA, 2-AG, HU210 and WIN55,212-2, as well as the enhancement of EC signaling with drugs that blocks ECs degradation (URB597 and URB574), increase cell proliferation [90, 92] whereas treatment with WIN55,212-2 and URB597 in CB_1_ knockout NPs failed to alter neurogenesis, indicating the requirement of CB_1_ receptors in cannabinoids induced NPs cell division [93]. Moreover, FAAH knockout mice, which present increased ECs levels, displayed a larger number of hippocampal BrdU+ cells [90]. On the same direction, studies *in vitro *showed that cannabinoid antagonists such as AM251, AM281, AM630, and the diacylglycerol lipase (DAGL) inhibitors RHC-80276 and THL, which decrease ECs biosynthesis, blocked the effect of cannabinoid agonists or decreased cell proliferation by themselves [92, 94]. 

Similar to CB_1_, CB_2_ receptors also seem to be involved in the modulation of adult hippocampal neurogenesis. Hippocampal NPs treated with the CB_2_ selective agonist HU-308 present increased cell proliferation whereas the CB_2 _antagonist SR144528 reduced neurogenesis [91, 95]. Interestingly, regulation of neurogenesis by DAGL-derived 2-AG has been shown to involve, at least in part, CB_2_ receptors [94].

### 3.3. Evidence from *in vivo* Studies

In accordance to these *in vitro *results, studies *in vivo *have also demonstrated the importance of the EC system to modulate cell proliferation, differentiation, maturation and survival. Moreover, there is a positive association between cannabinoid-induced neurogenesis and the behavioral improvement observed in animal models of anxiety, psychosis and depression. Chronic (10 days), but not acute, administration of HU210 induced anxiolytic- and antidepressive-like effects by increasing neurogenesis, once animals that were submitted to SGZ-x-ray did not show any behavioral response. Repeated administration of WIN55,212-2 was also able to promote cell division in mice and rats [92, 94, 96]. 

In addition to injections of exogenous agonists, the participation of ECs in the modulation of neurogenesis has also been investigated. Chronic treatment with URB597 (10 days) increased cell proliferation, while the ECs uptake inhibitor, AM404, reversed the trimethylthiazoline(TMT)-induced decrease of neurogenesis and inhibited defensive burying [94, 97].

Akin to the results observed with synthetic cannabinoids and ECs, two major constituents of the plant *Cannabis sativa*, the psychoactive compound delta-9-tetrahydrocannabinol (THC) and the non-psychoactive cannabidiol (CBD), may also affect adult hippocampal neurogenesis. Repeated treatment with CBD for 15-days prevented β-amyloid-induced neurotoxicity *via *activation of the proliferator-activated receptor-γ (PAAR-γ), suggesting a mechanism for CBD neuroprotective effects [98]. Also, CBD (42 days), despite decreasing cell proliferation, stimulated cell survival without promoting amelioration on spatial learning [99]. These responses were mediated by CB_1_ receptors, since CBD effects were absent in CB1-KO mice. More recently, a studied conducted with transgenic mice (GFAP-TK mice) showed that the anxiolytic effect of chronic CBD administration (14 days) in stressed mice depends on its proneurogenic action in the adult hippocampus by facilitating endocannabinoid-mediated signaling [100]. However it is important to stress that THC, a CB_1_ receptor partial agonist, can decrease cell proliferation and impair spatial memory [101]. In addition, Zhang and colleagues [101] have recently shown that mice lacking CB_1_ only on astrocytes were protected from memory impairments induced by high doses of THC, suggesting a THC mechanism independent of neuronal located CB1 receptors [101].

Similar to *in vitro *studies, CB_2_ was also shown to influence neurogenesis. Repeated administration of HU-308 during 5 days increased cell proliferation [91, 95], whereas the CB_2_ inverse agonist JTE907 or the antagonist SR144528 caused opposite results [94]. The involvement of CB_2_ receptors in these results was confirmed by the failure of the CB_2 _agonist to induce any change in CB_2 _deficient mice [95].

Although *in vitro* studies with NPs exposed to CB_1_ and CB_2 _antagonists/inverse agonists usually demonstrate unidirectional effect on neurogenesis, the use of these compounds *in vivo* shows contradictory results. While repeated administration of SR141716A and AM630 decreased neurogenesis in some studies [94, 102], Jin *et al*. [103] found that AM251 and SR141716A increased it, an effect present even in CB_1_-KO mice but absent in TRPV_1_-KO mice, suggesting the participation of the vanilloid system in the modulation of neurogenesis [94, 102]. These discrepancies may involve the animal species or gender used, the drug and BrdU treatment schedule, the drug dose and, importantly, the time-point where these measurements are performed, which may induce confusing interpretations. For example, Wolf *et al*. [99] found increased cell proliferation 1 and 24h after treatment with AM251, but a decrease in cell maturation 48h and 7 days later [99]. These results suggest that the role of cannabinoids on neurogenesis is complex and requires additional investigation.

## 4. ANTIDEPRESSANT TREATMENT MODULATES THE ENDOCANNABINOID SYSTEM

The putative role of cannabinoid in the control of mood and anxiety disorders has been describe by numerous authors [103, 104]. In addition, it has been suggested that the majority of the available treatments for depression modulates sendo-cannabinoid signaling. For instance, sleep deprivation, which can induce antidepressant effects, increases circulating levels of AEA in humans [103] and elevates 2-AG levels in the hippocampus [105]. A similar picture was found in the amygdala [106]. However, a decrease in CB_1_ receptor binding and in the amount of AEA in the prefrontal cortex was described by the same group [107]. Several studies have also provided evidence that chronic treatment with anti-depressant drugs such as SSRIs and tricyclic might modify the endocannabinoid system. For example, the tricyclic antidepressant desipramine, a noradrenergic uptake inhibitor, increases cannabinoid CB_1_ receptor density without changing endocannabinoid levels in the hypothalamus and hippocampus [107]. In addition, imipramine chronic treatment increases CB_1_ receptor binding in amygdaloid complex, but reduces CB_1_ receptor binding in the hypothalamus and striatum [108]. The SSRI fluoxetine increases the expression and promotes a facilitation of CB_1_ receptor mediated signaling in limbic areas such as the prefrontal cortex [109-111]. Conversely, in the study of Hesketh and colleagues [112], citalopram reduced CB_1_ mediated neurotransmission in the hippocampus and hypothalamus [112]. More recently however, it was shown that acute stimulation of CB_1_ receptors modulates the effect of citalopram on serotonin levels in the medial prefrontal cortex [113]. Regarding the monoamine oxidase (MAO) inhibitors, tranylcypromine reduced AEA content and increased CB_1_ receptor binding in the hippocampus and prefrontal cortex [110]. Even if there are contradictory results, in overall these findings support the hypothesis that the recruitment of the endocannabinoid system could be involved in the long lasting neuroplastic events (neurogenesis) promoted by AD chronic treatment.

Cannabinoids can also modulate serotonergic neurotransmission and serotonin subtypes 1A and 2A/2C receptor expression in the brain [114, 115]. Genetic deletion of the eCB degradation enzyme FAAH increases the firing of serotonergic neurons located in dorsal raphe nucleus. As a consequence, serotonin release is increased in limbic areas such as the prefrontal cortex [116]. Moreover, CB_1_ knockout mice displayed functional impairment of 5-HT_1A_ and 5-HT_2A/C_ receptor-mediated neurotransmission in the hippocampus [117] while a loss of antidepressants behavioral effects was described after genetic blocked of CB_1_ receptors [118].

Several studies point to an important bi-directional influence between the EC system and AD effects. For example, previous treatment with a CB_1_ receptor antagonist prevented the effects of imipramine on stress-induced activation of the hypothalamus-pituitary-adrenal axis [107]. Furthermore, treatment with the SSRI fluoxetine failed to facilitate serotonergic neurotransmission in the prefrontal cortex of CB_1_ knockout mice [119]. Likewise, long-term fluoxetine treatment up-regulated CB_1_ receptor signaling at the G protein transduction level in the prefrontal cortex [111].

However, even considering the possible role of neurogenesis facilitation by AD in their therapeutic effects [24, 28, 44, 120], no study, to our knowledge, has yet directly investigated if the disruption of the endocannabinoids system signaling could influence the pro-neurogenic effects of AD. Since facilitation of hippocampal endocannabinoid signaling (*via *CB_1_/CB_2_ receptor) is known to promote cell proliferation and neurogenesis [90, 91, 92, 94], and based on the evidence that AD treatment promotes changes in endocannabinoid signaling, it is possible that antidepressant chronic treatment modulates hippocampal neurogenesis *via *endocannabinoid system. 

**Fig. (1):**
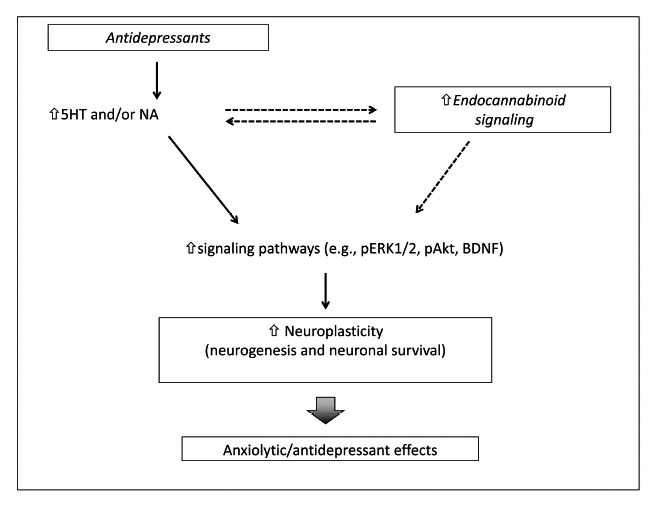
Interaction between antidepressants and the endocannabinoid system. Continuous arrows show proposed mechanisms of the neuroplastic hypothesis of antidepressant actions. Dashed arrows indicate possible interaction sites between antidepressant and endocannabinoids effects. 5HT: serotonin, NE: norepinephrine.

The results reviewed in the present paper so far suggest a common link between neurogenesis, antidepressant and endocannabinoids. Moreover, part of the positive effects of AD has also been related to changes in signaling pathways that regulate cellular plasticity and survival. Interestingly, a significant number of these intracellular pathways are also modulated by cannabinoid signaling. Long-term treatment with ADs up-regulates the cAMP-protein kinase A (PKA) and extracellular signal-regulated kinase (ERK) signaling pathway [117, 118]. Similarly, CB_1_ receptors are also coupled to ERK cascades and the proneurogenic action of cannabinoids seems to be related to facilitation of ERK signaling [91, 122, 123]. Also, brain derived neurotrophic factor (BDNF), a neurotrophin that is found reduced in depressed patients, and that is up regulated after AD or cannabinoids treatment could be involved [121, 124-127]. This neurotrophic factor has been implicated in adult hippocampal neurogenesis [128]. Activation of the BDNF receptor, TrkB, induces phosphorylation of ERK1/2 and Akt [129]. The Akt-mediated pathway is up regulated by dual reuptake inhibitor (SNRI) venlafaxine, which also facilitates hippocampal neurogenesis [130]. In a similar way, cannabinoids can increase *in vitro* neuroprogenitor cell proliferation by increasing the activation of the phosphatidylinositol 3-kinase/Akt signaling [93]. Therefore, additive or synergic effects on signaling pathways related to neurogenesis, cellular plasticity and survival mechanisms could be relevant for the endocannabinoids facilitatory effects on the therapeutic responses of ADs (Fig. **1**).

## 5. PERSPECTIVES AND CONCLUSIONS

The present paper reviewed the possible role of hippocampal neurogenesis on the behavioral effects of AD and cannabinoids. Several pieces of evidence support the proposal that the endocannabinoid signaling pathway could participate in behavioral actions of AD that may depend on hippocampal neurogenesis (Fig. **1**). In addition, disruption of endocannabinoid signaling by stressful situations could be involved in the stress-induced reduction of hippocampal neurogenesis. Additional studies, designed to test these possibilities, are needed to elucidate the role of the endocannabinoid system on the behavioral and pro-neurogenic effects of AD. 

## Figures and Tables

**Table 1.** Effect of Different Classes of Antidepressants on Adult Neurogenesis: *in vitro* and *in vivo* Studies

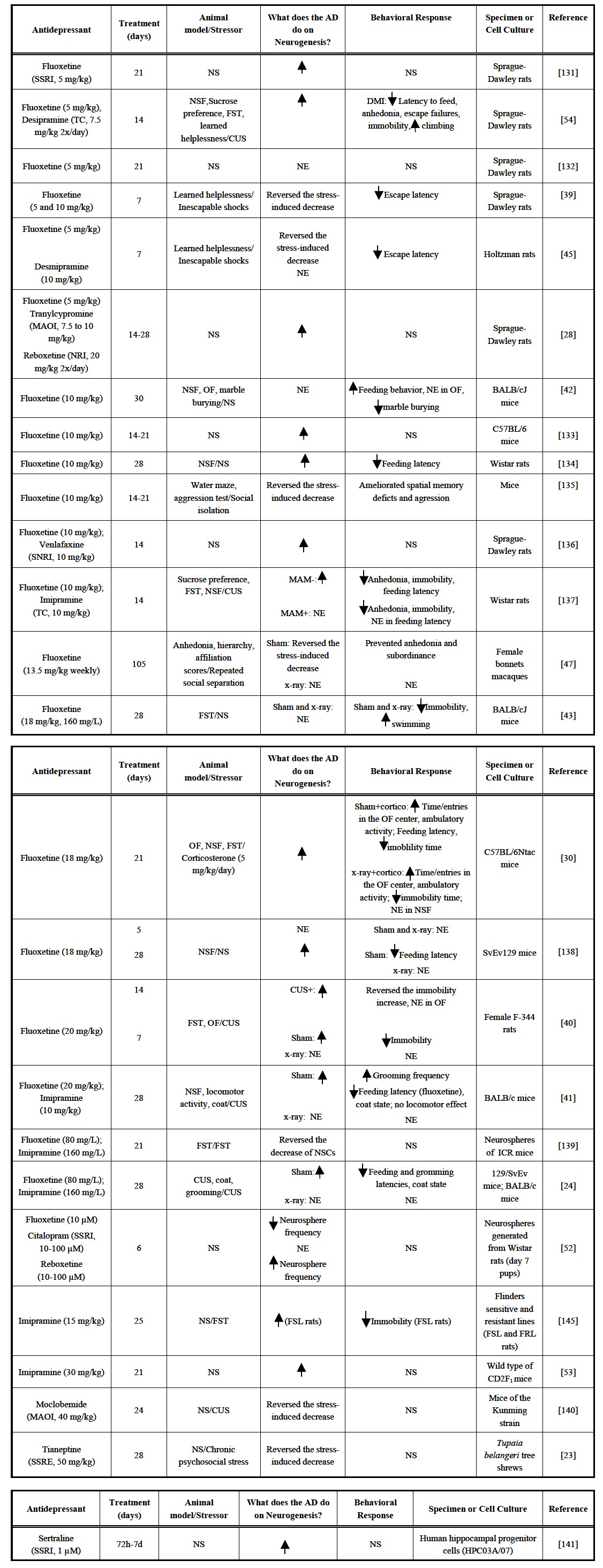

SSRI = selective serotonin reuptake inhibitor; TC = tricyclic; NRI = norepinephrine reuptake inhibitor; MAOI = monoamino oxidase inhibitor; SNRI = serotonin-norepinephrine reuptake inhibitor; SSRE = selective serotonin reubtake enhancer; NSF = novelty supressed feeding; FST = forced swimming test; OF = open field test; x-ray = x-irradiation of the subgranular zone of the dentate gyrus; sham = not irradiated animals; MAM = methylazoxymethanol; CUS = chronic unpredictable stress; NS = not studied; NE = no effect observed.

**Table 2.** Effect of Cannabinoids Compounds on Adult Neurogenesis: *in vitro* and *in vivo* Studies

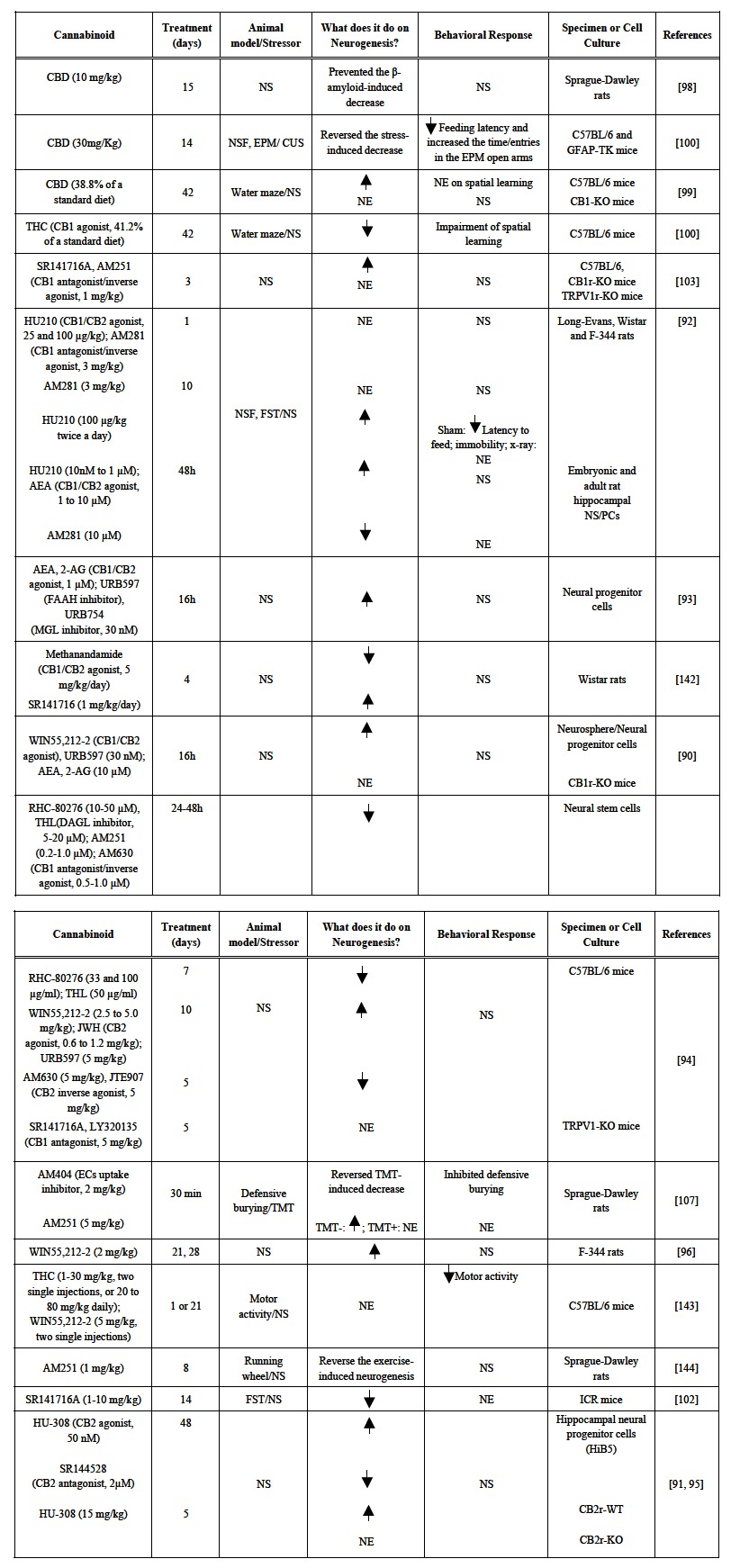

CBD = cannabidiol; AEA = anandamide; NSF = novelty supressed feeding; EPM= elevated plus maze FST = forced swimming test; x-ray = x-irradiation of the subgranular zone of the dentate gyrus; sham = not irradiated animals; TMT = trimethylthiazoline; NS = not studied; NE = no effect observed.
